# Cardiovascular disease behavioral risk factors among Latinos by citizenship and documentation status

**DOI:** 10.1186/s12889-020-08783-6

**Published:** 2020-05-06

**Authors:** Alexander N. Ortega, Jessie Kemmick Pintor, Brent A. Langellier, Arturo Vargas Bustamante, Maria-Elena De Trinidad Young, Michael L. Prelip, Cinthya K. Alberto, Steven P. Wallace

**Affiliations:** 1 Dornsife School of Public Health, Drexel University, Philadelphia, PA 19104 USA; 2 Fielding School of Public Health, University of California, Los Angeles, CA 90095 USA; 3 School of Social Sciences, Humanities and Arts, University of California, Merced, CA 95343 USA

**Keywords:** Immigrants, Hispanic Americans, Citizenship, Cardiovascular disease risk factors, Cardiovascular disease prevention, Heart disease

## Abstract

**Background:**

Studies have observed that recent Latino immigrants tend to have a physical health advantage compared to immigrants who have been in the US for many years or Latinos who are born in the United States. An explanation of this phenomenon is that recent immigrants have positive health behaviors that protect them from chronic disease risk. This study aims to determine if trends in positive cardiovascular disease (CVD) risk behaviors extend to Latino immigrants in California according to citizenship and documentation status.

**Methods:**

We examined CVD behavioral risk factors by citizenship/documentation statuses among Latinos and non-Latino US-born whites in the 2011–2015 waves of the California Health Interview Survey. Adjusted multivariable logistic regressions estimated the odds for CVD behavioral risk factors, and analyses were stratified by sex.

**Results:**

In adjusted analyses, using US-born Latinos as the reference group, undocumented Latino immigrants had the lowest odds of current smoking, binge drinking, and frequency of fast food consumption. There were no differences across the groups for fruit/vegetable intake and walking for leisure. Among those with high blood pressure, undocumented immigrants were least likely to be on medication. Undocumented immigrant women had better patterns of CVD behavioral risk factors on some measures compared with other Latino citizenship and documentation groups.

**Conclusions:**

This study observes that the healthy Latino immigrant advantage seems to apply to undocumented female immigrants, but it does not necessarily extend to undocumented male immigrants who had similar behavioral risk profiles to US-born Latinos.

## Background

Cardiovascular disease (CVD) accounts for 20% of deaths among Latinos [1]. Latinos have disparities in clinical (e.g., blood pressure, BMI) and behavioral (e.g., fast food and soda consumption, fruits/vegetable intake, exercising, and blood pressure medication use) risk factors for CVD [2, 3], although there is significant within group variability [4, 5]. Research has observed variations among Latinos in CVD risk factors and outcomes based on factors such as sex, nativity, language use, time in the United States (US) and generational status [4, 5]. One factor that has not been well studied in the CVD and Latino health literature is citizenship and documentation status and its association with CVD risk factors and outcomes. Citizenship and documentation status (i.e., undocumented, documented, naturalized citizen) influence immigrants’ migration and integration trajectories in the US [6], which may potentially influence the behavioral and environmental factors that increase the risk of CVD. Documentation status is particularly salient since the majority (66%) of undocumented immigrants have been in the US for more than 10 years [7], and as they get older their risk for CVD will increase.

Latino immigrant documentation status is associated with access to and utilization of health care, with studies showing undocumented Latino immigrants having lower rates of insurance, usual sources of care and use of health services than other US-born or immigrant Latinos [8–11] Having access to primary care has been shown to be positively associated with CVD behavioral prevention among Latinos [12]. Using a Los Angeles-based survey that included in-person blood pressure measurements, Young and Pebley (2017) found that undocumented Latino immigrants had higher rates of high blood pressure than documented Latino immigrants, and they had similar rates of high blood pressure to those who were US-born [13]. Moreover, while documented immigrants who had been in the US for less than 15 years had lower rates of high blood pressure compared to their documented counterparts who had been in the country more than 15 years, they found that time in the US was not associated with blood pressure for undocumented immigrants [13]. These findings suggest that there was not a healthy immigrant effect for undocumented immigrants. A recent study using pooled 2011–2015 waves of the California Health Interview Survey (CHIS) found that while undocumented Latinos had the worst patterns of access to and use of health services, they had better self-reported CVD clinical risk profiles, such as lower rates of overweight/obesity, high blood pressure and diabetes compared with other Latino immigrants and those who were US-born [10].

To our knowledge, there has been no research on the associations among citizenship and documentation status and CVD behavioral risk factors among Latinos using a large statewide representative sample. Thus, this study examines whether self-reported CVD behavioral risk factors varied by citizenship and documentation statuses among Latinos in California. Using pooled 2011–2015 waves of CHIS, which is the largest state-level health and health care survey in the US and one of the only population-based, representative surveys with measures of immigrant documentation statuses, we examine the relationships among citizenship and documentation statuses (i.e., US-born, naturalized, green card holder, undocumented) and CVD behavioral risk factors (i.e., smoking, binge drinking, fast food and soda consumption, fruits/vegetable intake, walking, and blood pressure medication use) for nonelderly Latino adults.

## Methods

### Study population and data source

We use data from the 2011–2015 waves of the California Health Interview Survey (CHIS). CHIS is a representative, cross-sectional survey of the noninstitutionalized population in California. The survey is administered in five languages including Spanish and is conducted via a dual-frame random-digit-dial design (both landline and cellphone). Since 2011, CHIS has been an ongoing survey with data collected continuously during a two-year data collection cycle. We limit our sample to nonelderly (ages 18–64 years) Latino and US-born non-Latino white adults for a total sample of 51,386 participants. More details on CHIS are available elsewhere [14].

### Measures

#### Citizenship and documentation status

CHIS is the only state-level population-based representative survey which measures both citizenship and documentation statuses for immigrants. CHIS asks participants about their race, Latino ethnicity and nativity. To determine citizenship status, CHIS asks participants who are foreign-born “are you a citizen of the United States?” Citizens are then asked, “when did you become naturalized?,” while noncitizens are asked, “are you a permanent resident with a green card?” Following previous peer-reviewed studies [9–11, 15–17], immigrant participants who are noncitizens and do not have a green card are classified as undocumented. We use information on citizenship and documentation statuses to create the following mutually exclusive groups for Latinos: US-born (8303), naturalized (3879), green card (3369) undocumented (3053). We also include non-Latino US-born whites as a reference group (32,782). A previous analysis of CHIS data found that using the “no green card” status as a proxy for “undocumented” and relying on self- reports for green card and naturalized citizenship status is valid and results in only a 5% misclassification error [18].

#### CVD behavioral risk factors

We measured CVD behavioral risk factors through the following CHIS measures: current smoking status (current smoker, currently not smoking), having engaged in binge drinking in the past year (5 or more drinks for males on one occasion, 4 or more drinks for females on one occasion), having eaten fast food in the past week, the total number of times ate fast food in the past week, having drunk soda at least once in the past week, the total number of times drank soda in the past week, having eaten fruits or vegetables at least 5 times per day in the past week, the number of times the participant walked for 10 min or more for leisure in the past week, and, for those with a physician diagnosis of high blood pressure, if the participant was currently taking blood pressure medication. The “having eaten fruits or vegetables at least 5 times per day in the past week” measure was derived from a weekly measure. We summed the weekly consumption of fruits and vegetables and then divided by seven to obtain a daily measure. We chose five as the threshold since the United States Department of Agriculture recommends that adults should consume 1.5–2.0 cups of fruit and 2.0–3.0 cups of vegetables per day [19].

Independent Measures.

Our independent measures include sex (male, female), marital status (married, not married), age (18–34, 35–49, 50–64), English language proficiency (speaks only English, very well or well; speaks English not well or not at all), employment status (currently employed, not currently employed), income as a percentage of the federal poverty level (FPL) (0–138%, 139–250%, 251–400%, ≥400%), location of residence (urban, not urban), education (less than high school, high school, more than high school), insurance status (currently insured, currently uninsured), self-reported health status (poor, fair, good, very good, excellent) and survey year.

#### Statistical analyses

To account for the complex sampling design of CHIS and for non-response, we used survey weights and design variables in all analyses. First, we conducted chi-squared analyses to assess variation across citizenship and documentation statuses and our independent measures. Second, we conducted chi-squared and analysis of variance (ANOVA) analyses to assess significant variations across citizenship and documentation statuses and our CVD behavioral risk factor measures. Given previously observed differences in CVD risk factors by sex among Latinos [4, 5], the second set of analyses were stratified by sex (male/female). Lastly, we ran multivariable regression analyses to assess the associations among citizenship and documentation status and CVD behavioral risk factors, adjusting for the independent variables. Binary outcomes were modeled using multivariable logistic regressions, and count outcomes were modeled using multivariable Poisson regression. We report odds ratios (ORs) and 95% confidence intervals (CIs) for all logistic regressions and incidence rate ratios (IRRs) and 95% CI’s for Poisson regressions. Stata, version 14, was used for the statistical analyses.

## Results

In Table 1, we showed the sample characteristics for Latinos by citizenship and documentation status and for non-Latino whites. Undocumented Latino immigrants had the highest proportion of any documentation status group of younger adults, the lowest proportion who speak English well or very well, the highest proportion under 138% of FPL, the lowest levels of education and lowest proportion with health insurance. The non-citizen groups were similar in having low proportions of respondents who reported excellent or very good health status.

**Table 1:** Descriptive Statistics by Race/Ethnicity and Citizen and Documentation Status, California Residents Ages 18–64, 2011–2015

	Total (*N* = 51,386)	Latino (*n* = 18,604)				Non-Latino White	*p*-value^a^
		US-born (*N* = 8303)	Naturalized Citizens (*N* = 3879)	Green Card Holders (*N* = 3369)	Undocumented Residents (*N* = 3053)	US-born (*N* = 32,782)	
Female	49.70	49.28	53.91	49.40	48.84	49.29	0.0226
Married	49.20	31.30	67.65	61.32	43.57	53.03	< 0.001
Age							
18–34	37.76	63.05	17.21	25.21	37.07	32.13	< 0.001
35–49	32.43	22.36	40.36	45.39	52.48	29.33	
50–64	29.81	14.59	42.43	29.41	10.45	38.55	
Speaks English Well or Very Well	81.20	97.99	57.52	30.58	16.85	99.94	< 0.001
Currently Employed	72.22	69.65	72.51	71.16	69.13	74.22	< 0.001
FPL							
0–138	28.81	29.69	35.71	54.64	71.99	13.67	< 0.001
139–250	18.21	22.38	27.55	27.61	19.14	12.42	
251–400	16.80	19.95	19.54	10.43	6.25	17.99	
400 or above	36.17	27.99	17.20	7.33	2.62	55.92	
Urban Area	88.58	92.37	93.18	88.64	92.20	85.09	< 0.001
Education							
Less Than High School	19.30	9.04	39.46	58.15	64.63	4.06	< 0.001
High School	25.03	35.61	26.57	21.06	22.99	20.73	
More than High School	55.67	55.35	33.97	20.78	12.38	75.21	
Currently Insured	80.13	79.91	78.62	68.21	46.84	89.36	< 0.001
Self-Reported Health							
Excellent	18.95	17.29	15.96	10.45	10.44	23.61	< 0.001
Very Good	30.17	31.83	19.75	12.85	14.55	37.75	
Good	31.11	32.65	36.00	38.66	39.36	26.35	
Fair	16.26	14.20	24.00	33.77	32.30	9.30	
Poor	3.51	4.04	4.29	4.27	3.36	2.99	
Survey Year							
2011	19.75	19.29	20.39	18.98	16.15	20.70	0.001
2012	19.91	19.12	20.59	20.92	20.03	19.94	
2013	20.03	21.26	18.18	19.22	20.52	19.86	
2014	20.09	20.41	21.04	21.55	19.30	19.64	
2015	20.22	19.91	19.80	19.33	24.00	19.86	

Unless otherwise indicated data are expressed as column percentages

^a^ Calculated by means of χ^2^ tests across Latino and US-born non-Latino white samples

In Table 2, we showed CVD behavioral risk factors stratified by sex for Latinos by citizenship and documentation status and for non-Latino whites.

**Table 2:** CVD Behavioral Risk Factors by Race/Ethnicity and Citizenship and Documentation Status, California Residents Ages 18–64, 2011–2015

	Total (*N* = 51,386)	Latino (*n* = 18,604)				Non-Latino White	*p*-value^a^
		US-born (*N* = 8303)	Naturalized Citizens (*N* = 3879)	Green Card Holders (*N* = 3369)	Undocumented Residents (*N* = 3053)	US-born (*N* = 32,782)	
Current Smoker							
Total	14.28	13.00	10.06	12.88	11.05	16.66	< 0.001
Male	17.45	15.54	15.43	19.79	18.51	18.11	0.891
Female	11.08	10.38	5.46	5.79	3.23	15.17	< 0.001
Binge Drinking in Past Year							
Total	39.73	44.51	29.45	33.32	30.63	42.48	< 0.001
Male	47.52	49.77	43.21	50.54	43.44	47.5	0.0132
Female	31.84	39.09	17.68	15.68	17.22	37.32	< 0.001
Ate Fast Food at Least Once in Past Week							
Total	69.02	80.31	69.61	70.02	72.63	62.54	< 0.001
Male	71.85	81.25	72.02	73.54	72.89	66.76	< 0.001
Female	66.15	79.36	67.54	66.41	72.36	58.19	< 0.001
Number of Times Ate Fast Food Last Week							
Total	1.61	2.25	1.47	1.74	1.77	1.41	< 0.001
Male	2.09	2.54	1.89	2.29	2.16	1.85	< 0.001
Female	1.37	1.37	1.34	1.37	1.27	1.49	< 0.001
Drank Soda at Least Once in Past Week							
Total	47.55	55.94	52.21	61.21	67.38	36.31	< 0.001
Male	55.99	62.23	62.42	70.43	74.97	45.35	< 0.001
Female	39.01	49.46	41.63	51.77	59.44	27.01	< 0.001
Number of Times Drank Soda Last Week							
Total	1.91	2.64	1.82	3.04	3.49	1.50	< 0.001
Male	3.45	3.38	2.73	4.16	4.73	2.23	< 0.001
Female	1.80	2.16	1.26	2.14	2.21	1.23	< 0.001
Ate Fruits or Vegetables at Least 5 Times Per Day^b^							
Total	7.39	5.17	4.75	5.22	4.66	9.71	< 0.001
Male	5.58	5.01	4.00	1.41	2.96	7.25	0.0036
Female	9.24	5.33	5.41	9.38	6.32	12.21	< 0.001
Number of Times Walked for at Least 10 Minutes Last Week							
Total	4.30	4.07	4.08	3.85	3.75	4.56	< 0.001
Male	4.00	4.22	3.77	3.71	3.77	4.51	< 0.001
Female	3.99	3.89	4.09	3.87	3.73	4.37	< 0.001
Currently Taking Blood Pressure Medication (*n* = 13,776)							
Total	55.42	50.47	59.77	53.73	32.96	60.44	< 0.001
Male	54.70	47.74	57.19	51.84	38.19	59.94	< 0.001
Female	56.23	54.02	62.09	55.61	27.80	61.03	< 0.001

Unless otherwise indicated data are expressed as column percentages

^a^ Calculated by means of χ^2^ and analysis of variance tests across Latino and US-born non-Latino white samples

^b^Derived from a weekly measure

### Smoking and binge drinking

Undocumented Latino immigrants were overall less likely to be current smokers than all other groups except naturalized immigrants. There were no differences in current smoking by documentation status for men and, among women, undocumented immigrants were the least likely to currently smoke. Overall and among men, undocumented and naturalized Latino immigrants were the least likely to report binge drinking in the last year, and women holding green cards had the lowest proportion of those reporting binge drinking among women.

### Fast food, soda and fruit and vegetable intake

Any fast food consumption in the past week was reported by a lower proportion of all immigrant groups than US-born Latinos, although at a higher proportion than US-born whites, with similar patterns for men and women. The number of times in the past week respondents ate fast food was lower among all groups of immigrant men than US-born Latino men and higher than US-born white men. Among women, however, undocumented immigrants had the lowest average frequency of eating fast food in the past week across all groups. Undocumented immigrants also had the highest proportion of having drank soda at least once in the past week and having drank soda the highest number of times in the past week; this was consistent for men and women. Undocumented immigrants had the lowest proportion reporting that they ate fruits and vegetables at least 5 times per day. While undocumented immigrant men were less likely to report eating fruits and vegetables at least 5 times per day than all other groups, undocumented women were more likely to have eaten fruits and vegetables than their US-born and naturalized counterparts.

### Leisure walking

Overall, undocumented Latino immigrants and green card holders reported the lowest frequency of leisure walking of at least 10 min in the past week compared to the other groups. Across the immigrant groups, men had similar frequencies of leisure walking, and undocumented female immigrants had the lowest frequency of all groups.

### Blood pressure medication

Among those with a physician diagnosis of high blood pressure, undocumented immigrants had much lower rates of currently taking blood pressure medication, and this disparity was even more pronounced for women.

### Adjusted multivariable regressions

When controlling for the independent measures, undocumented immigrants had better CVD risk behaviors than US-born Latinos in three of eight categories (smoking, binge drinking, and number of times ate fast food) (Table 3). There were no categories where undocumented immigrants had worse risk profiles than US-born Latinos. In the stratified analyses, undocumented men had better behaviors only in number of times ate fast food, while undocumented women had lower odds of smoking, binge drinking, number of times ate fast food, and number of times having drank soda. Undocumented women who reported hypertension had a substantially lower odds of taking blood pressure medication than US-born Latinos, which was the only negative comparison among all the stratified comparisons.

**Table 3:** Multivariable Analyses of CVD Behavioral Risk Factors by Race/Ethnicity and Citizenship and Documentation Status, California Residents Ages 18–64, 2011–2015

	Model	Latino (*n* = 18,604)				Non-Latino White
		US-born	Naturalized Citizens	Green Card Holders	Undocumented Residents	US-born
Current Smoker						
All	Logistic (OR) [CI]	REF	0.82 [0.63, 1.05]	0.90 [0.68, 1.21]	0.61** [0.46, 0.81]	2.02*** [1.74, 2.35]
Men	Logistic (OR) [CI]	REF	1.08 [0.77, 1.52]	1.17 [0.82, 1.68]	0.81 [0.57, 1.16]	1.86*** [1.55, 2.24]
Women	Logistic (OR) [CI]	REF	0.55** [0.37, 0.82]	0.58 [0.33, 1.01]	0.27*** [0.16, 0.47]	2.26*** [1.82, 2.81]
Binge Drinking in Past Year						
All	Logistic (OR) [CI]	REF	0.86 [0.74, 1.01]	0.99 [0.83, 1.18]	0.75** [0.62, 0.91]	1.15** [1.06, 1.25]
Men	Logistic (OR) [CI]	REF	1.15 [0.92, 1.44]	1.42** [1.10, 1.81]	0.87 [0.66, 1.14]	1.15* [1.01, 1.30]
Women	Logistic (OR) [CI]	REF	0.62*** [0.49, 0.79]	0.60*** [0.45, 0.78]	0.62** [0.45, 0.85]	1.15*** [1.01, 1.31]
Fast Food at Least Once in Past Week						
All	Logistic (OR) [CI]	REF	0.80* [0.66, 0.95]	0.81* [0.66, 1.00]	0.84 [0.67, 1.05]	0.55*** [0.50, 0.62]
Men	Logistic (OR) [CI]	REF	0.84 [0.64, 1.11]	0.92 [0.68, 1.24]	0.83 [0.59, 1.17]	0.61*** [0.52, 0.72]
Women	Logistic (OR) [CI]	REF	0.76* [0.61, 0.94]	0.71* [0 .53, 0.96]	0.85 [0.61, 1.17]	0.51*** [0.44, 0.58]
Number of Times Ate Fast Food Last Week						
All	Poisson (IRR) [CI]	REF	0.82*** [0.76, 0.90]	0.90* [0.81, 1.00]	0.82*** [0.73, 0.91]	0.79*** [0.74, 0.83]
Men	Poisson (IRR) [CI]	REF	0.85** [0.76, 0.95]	0.97 [0.85, 1.11]	0.83* [0.71, 0.97]	0.85*** [0.79, 0.92]
Women	Poisson (IRR) [CI]	REF	0.79*** [0.71, 0.88]	0.80** [0.70, 0.93]	0.79*** [0.70, 0.90]	0.71*** [0 .65, 0.77]
Drank Soda at Least Once in Past Week						
All	Logistic (OR) [CI]	REF	1.00 [0.85, 1.17]	1.11 [0.93, 1.33]	1.14 [0.93, 1.39]	0.67*** [0.61, 0.74]
Men	Logistic (OR) [CI]	REF	1.17 [0.92, 1.50]	1.29* [1.00, 1.66]	1.29 [0.96, 1.73]	0.74*** [0 .65, 0.85]
Women	Logistic (OR) [CI]	REF	0.87 [0.70, 1.07]	0.96 [0.75, 1.22]	0.98 [0.75, 1.28]	0.61*** [0.53, 0.69]
Number of Times Drank Soda Last Week (Count - Poisson IRR)						
All	Poisson (IRR) [CI]	REF	0.73*** [0.63, 0.85]	0.93 [0.79, 1.10]	0.85 [0.73, 1.00]	0.89* [0.81, 0.99]
Men	Poisson (IRR) [CI]	REF	0.80* [0.66, 0.96]	0.98 [0.79, 1.22]	0.92 [0.74, 1.13]	0.90 [0.80, 1.02]
Women	Poisson (IRR) [CI]	REF	0.63*** [0.50, 0.80]	0.86 [0.68, 1.09]	0.74* [0.58, 0.95]	0.89 [0.75, 1.05]
Ate Fruits or Vegetables at Least 5 Times Per Day^a^						
All	Logistic (OR) [CI]	REF	0.89 [0.53, 1.52]	1.04 [0.59, 1.85]	1.01 [0.48, 2.15]	1.73*** [1.28, 2.33]
Men	Logistic (OR) [CI]	REF	0.75 [0.29, 1.92]	0.24** [0.09, 0.63]	0.50 [0.12, 1.98]	1.51 [0.96, 2.37]
Women	Logistic (OR) [CI]	REF	1.06 [0.57, 1.97]	2.10* [1.08, 4.10]	1.67 [0.73, 3.82]	1.93** [1.31, 2.84]
Number of Times Walked for at Least 10 Minutes Last Week (Count - Poisson IRR)						
All	Poisson (IRR) [CI]	REF	0.98 [0.89, 1.08]	0.97 [0.89, 1.04]	0.96 [0.88, 1.04]	1.07* [1.01, 1.13]
Men	Poisson (IRR) [CI]	REF	0.91 [0.83, 1.00]	0.93 [0.84, 1.04]	0.95 [0.86, 1.05]	1.05 [0.97, 1.14]
Women	Poisson (IRR) [CI]	REF	1.04 [0.91, 1.20]	1.00 [0.91, 1.12]	0.98 [0.87, 1.11]	1.08 [1.00, 1.16]
Currently Taking Blood Pressure Medication (*n* = 13,776)						
All	Logistic (OR) [CI]	REF	0.73 [0.52, 1.02]	0.83 [0.53, 1.29]	0.59* [0.36, 0.95]	0.83 [0.67, 1.03]
Men	Logistic (OR) [CI]	REF	0.66 [0.40, 1.08]	0.82 [0.41, 1.658]	0.99 [0.49, 2.00]	0.80 [0.60, 1.06]
Women	Logistic (OR) [CI]	REF	0.76 [0.47, 1.23]	0.82 [0.46, 1.48]	0.35** [0.18, 0.67]	0.87 [0.63, 1.20]

Adjusted for sex, marital status, health insurance, age, education, English language proficiency, employment, federal poverty level, urban/rural area, self-reported health, and survey year

^a^Derived from a weekly measure

CI denotes a 95% confidence interval; OR denotes an odds ratio; IRR denotes an incidence rate ratio

***p < 0.001, **p < 0.01, *p < 0.05

Compared to US-born Latinos, US-born non-Latino whites had higher odds of smoking and binge drinking and lower odds of eating any/more often fast food, drinking any/more often soda and higher odds of fruit/vegetable consumption and leisure walking (Table 3).

## Discussion

To our knowledge, this is the first study to examine the relationships among citizenship and documentation statuses and CVD behavioral risk factors for Latinos using a large, state-level representative survey. Our findings suggest that the behavioral risk factors of undocumented Latino immigrants are sometimes better and rarely worse when compared with US-born Latinos, while non-Latino whites are sometimes better and sometimes worse than US-born Latinos. The most important contribution of this study is showing that most of the favorable risk factor trends among undocumented residents are driven by the better odds for women but not for men, with the exception of hypertension treatment. These findings are consistent with those of Young and Pebley (2017), who observed that there was not a healthy immigrant effect for high blood pressure for undocumented Latino immigrants [13].

The demographic profile of undocumented Latino immigrants has changed over the past three decades. In 1995, 33% of undocumented immigrants had been in the US for more than ten years, and in 2016 that percent rose to 66% [7]. Also, recent Latino immigrants are more likely to come from urban areas in Latin America and from Central America than in the past [20]. In the US, over 70% of undocumented immigrants are Latino, and 51% of undocumented Latino immigrants come from Mexico [21, 22]; this percent is even higher in California, where 69% of undocumented immigrants are from Mexico [21]. In our study, while not significant in the adjusted analysis, undocumented Latino immigrants were the most likely to report drinking soda in the past week in the unadjusted analysis; this unadjusted finding might reflect longstanding consumption patterns in Mexico, where sugar-sweetened beverage consumption is high [23] and contributes 69% of added sugars in the Mexican diet [24]. Sweetened beverage consumption in Mexico, as well as the high rate of obesity (33% in Mexico compared to 40% in the US) [25], prompted the country to pass a one-peso-per-liter tax that has resulted in a decrease in consumption by up to 10% [26]. This decreased consumption among the Mexican population may not “spill over” to Latino immigrants in the US, because most immigrants have lived in the US for more than a decade, and migration from Mexico into the US is low relative to the size of the existing immigrant population from Central America [7, 27]. Of course, this does not explain why green card holders have the lowest proportion reporting having drank soda. The relationship between diet and documentation status is likely complex and involves multiple levels of influence including living in enclaves where access to healthy foods is poor [28, 29].

The frequency of fast food consumption in the past week was lower for all immigrants versus US-born Latinos. This is likely due to the low level of consumption of food prepared away from home in Mexico and other Latin American countries [30]. Consumption and spending on food prepared away from home are particularly low among Mexicans with demographic characteristics similar to those of migrants, particularly those from rural areas and with low levels of income and education. In fact, the positive relationship between socioeconomic status and food prepared away from home among the Mexican population mirrors our findings of higher fast food consumption among US-born Latinos than among immigrants. We did not find any differences across the groups for fruit and vegetable consumption.

Undocumented Latinos were the only immigrant group that had lower odds of current smoking and binge drinking compared with US-born Latinos. This is consistent with the findings of a claims-based study in Southern California that found low rates of smoking and drinking among undocumented immigrants in a county health system [31]. While we did not find differences in leisure walking, it should be noted that this measure does not account for other forms of physical activity such as work-related physical activity (e.g., farming, construction) or transportation.

A recent paper showed that the Patient Protection and Affordable Care Act (ACA) reduced hypertension treatment disparities for Mexican-heritage Latinos. That study, however, did not look at the effects among Latino immigrant groups according to citizenship or documentation status [32]. Our findings show that undocumented immigrants who self-reported a physician diagnosis of high blood pressure were the least likely to be taking blood pressure medication compared with US-born Latinos, even after adjusting for health insurance.

Other studies have found that CVD risk profiles for Latinos vary by sex [4, 5]. In our study, we found that sex moderated some associations between citizenship and documentation statuses and CVD behavioral risk factors, such as current smoking and binge drinking, where undocumented immigrant Latinas had lower rates than US-born Latinas. Undocumented immigrant Latinas had lower frequency of soda consumption in past week and lower odds of taking blood pressure medication among those with high blood pressure than US-born Latinas. There were no differences for men.

Our study has limitations that should be noted. First, we were limited to the CVD behavioral risk factor measures available in CHIS. While these measures assess behavioral risk factors related to diet, smoking, and exercise, they are not necessarily comprehensive. Second, as in most observational studies that rely on participants to recall behaviors, our measures are subject to recall bias. Third, our documentation status measure is based on participants reporting whether they were foreign-born and having a green card or being naturalized, which may result in some misclassification, particularly for international students and those with temporary visas. Nevertheless, any misclassification would result in more conservative estimates for undocumented immigrants. Finally, our observations are limited to California. While California has the largest undocumented immigrant population compared with other states, our findings may not be entirely generalizable to other parts of the country.

## Conclusions

This study shows that documentation status is important to consider when studying heart disease prevention for Latinos. To the extent that men are more likely to suffer from CVD, and undocumented Latino men have behavioral risk profiles similar to US-born Latinos, we would not expect to see better CVD outcomes for undocumented immigrants than the US-born. The pattern is similar among Latino green card holders and naturalized citizens compared to US-born Latinos, which indicates that there is little or no immigrant health advantage in CVD risk among men, but there likely is among women. Given that undocumented Latino immigrants also have significant barriers to health care, including insurance eligibility, access to health care providers, and language concordant care [9–11], future studies should be cognizant of citizenship and documentation statuses when identifying clinical interventions to reduce CVD outcomes.

## Data Availability

The data that support the findings of this study are available from the UCLA Center for Health Policy Research, but restrictions apply to the availability of secured data. Public use data are available to the public, and use of secured data is provided by application and permission from the UCLA Center for Health Policy Research. Instructions on how to apply for accessing secured CHIS data can be found here: https://healthpolicy.ucla.edu/chis/data/Pages/confidential.aspx.

## Abbreviations

ACA: Affordable Care ActANOVA: Analysis of Variance
CHIS: California Health Interview Survey
CI: Confidence Intervals
CVD: Cardiovascular Disease
FPL: Federal Poverty Level
IRR: Incidence Rate Ratios
OR: Odds Ratio
US: United States
